# Radiological predictors of PCP in HIV-positive adults in South Africa: A matched case-control study

**DOI:** 10.4102/sajhivmed.v25i1.1636

**Published:** 2024-11-08

**Authors:** Nicola K. Wills, Jared Tavares, Qonita Said-Hartley, Sean Wasserman

**Affiliations:** 1 Department of Medicine, Faculty of Health Sciences, University of Cape Town, Cape Town, South Africa; 2 Department of Statistics, Faculty of Science, University of Cape Town, Cape Town, South Africa; 3 Department of Radiology, Faculty of Health Sciences, University of Cape Town, Cape Town, South Africa; 4 Institute for Infection and Immunity, St George’s University of London, London, United Kingdom; 5 Centre for Infectious Diseases Research in Africa, Institute of Infectious Disease and Molecular Medicine, University of Cape Town, Cape Town, South Africa; 6 MRC Centre for Medical Mycology, Faculty of Health and Life Sciences, University of Exeter, Exeter, United Kingdom

**Keywords:** HIV, PCP, *Pneumocystis jirovecii*, chest X-ray, prediction rule

## Abstract

**Background:**

Definition of chest X-ray (CXR) features associated with laboratory-confirmed pneumocystis pneumonia (PCP) among HIV-positive adults is needed to improve diagnosis in high-burden settings.

**Objectives:**

Our primary objective was to identify CXR features associated with confirmed PCP diagnosis and severe PCP (defined by hypoxia, intensive care unit referral/admission, and/or in-hospital death). We also explored the performance of logistic regression models, incorporating selected clinical and CXR predictors, for PCP diagnosis and severe PCP.

**Method:**

We conducted a case-control study involving HIV-positive adults with laboratory-confirmed PCP and a matched cohort with non-PCP respiratory presentations at regional hospitals in Cape Town, South Africa (2012–2020).

**Results:**

Records from 104 adults (52 PCP cases and 52 non-PCP controls) were included. Diffuse versus patchy ground-glass opacification was associated with increased odds of PCP diagnosis (adjusted odds ratio [aOR]: 6.2, 95% confidence interval [CI]: 1.6–28.9, *P* = 0.01) and severe PCP (aOR: 4.5, 95% CI: 1.6–14.4, *P* = 0.008). Consolidation was associated with severe PCP (aOR: 3.3, 95% CI: 1.2–11.0, *P* = 0.03) as was increasing ground-glass zone involvement (aOR: 2.1 for each one-unit increase in involved zone; 95% CI: 1.4–3.2, *P* = 0.0004). Models incorporating hypoxia (hypoxia model) or tachypnoea (respiratory rate model) with diffuse ground-glass opacities, absence of pleural effusion or reticular/reticulonodular changes on CXR performed well in predicting PCP (area under the receiver operating characteristic curve 0.828 [hypoxia model] and 0.857 [respiratory rate model]).

**Conclusion:**

CXR evaluation alongside bedside clinical information offers good accuracy for discriminating definite PCP from other HIV-associated respiratory diseases.

**What this study adds:** In this case-control study of 52 cases of HIV-associated PCP matched to 52 cases of non-PCP respiratory disease, we found diffuse ground-glass CXR changes to correlate with PCP diagnosis and severe PCP. Clinical prediction models, incorporating respiratory rate or hypoxia with select CXR features, showed good accuracy for predicting PCP.

## Introduction

Pneumocystis pneumonia (PCP), caused by the ubiquitous fungus, *Pneumocystis jirovecii*, is a common and severe HIV-associated opportunistic infection that has an estimated case-fatality rate of 19% amongst HIV-positive adults in sub-Saharan Africa.^1^ The lack of validated clinical definitions of PCP, frequent co-infections, and limited access to costly and invasive laboratory diagnostics complicates PCP diagnosis and may contribute to its poor treatment outcomes in high-burden settings. Chest X-ray (CXR) offers a potentially cost-effective,^2^ widely available and non-invasive diagnostic tool to more rapidly identify patients with PCP in resource-limited healthcare settings. However, CXR features that are predictive of laboratory-confirmed PCP, as opposed to other common causes of respiratory presentations among HIV-positive adults, have not been rigorously evaluated, limiting the utility of CXR for clinical decision-making. A meta-analysis of CXR patterns associated with presumptive PCP in HIV-positive adults in low- and middle-income countries highlighted the potential diagnostic value of CXR. However, in that review, clinical diagnostic definitions with low specificity were employed, and an analysis of the diagnostic predictive value of individual CXR features was not performed.^3^

Chest X-ray features that are associated with severe HIV-associated PCP would be helpful for early stratification of patients who may require escalated care, but have not been well described. Furthermore, whilst clinical prediction models for PCP diagnosis have been explored in South African^4^ and other settings,^5,6,7^ these have not incorporated specific PCP-associated CXR features alongside objective clinical determinants, which may improve diagnostic performance.

We aimed to better define CXR features that discriminate HIV-associated PCP from other common respiratory presentations in a high-burden setting, and to assess the performance of a prediction model incorporating significant radiological features and bedside clinical parameters for PCP diagnosis and severity.

## Research methods and design

### Study design

We conducted a case-control study, extracting clinical and radiological data from medical records of HIV-positive adults (≥ 18 years) admitted with respiratory disease and undergoing *P. jirovecii* respiratory sample testing at Western Cape hospitals between 2012 and 2020. Primary objectives were to explore CXR features associated with PCP diagnosis and severe PCP (as defined by marked hypoxia, intensive care unit [ICU] referral or admission, or in-hospital death). As secondary objectives, we explored the performance of a model, incorporating *a priori* and identified clinical and radiological features, to predict HIV-associated PCP diagnosis and severity.

### Study population and data sources

Definite PCP (cases) and non-PCP respiratory disease (controls) was assigned using pre-specified diagnostic criteria, adapted from Centers for Disease Control and Prevention (CDC) and World Health Organization (WHO) guidelines (Table 1). PCP cases were matched to controls based on most recent CD4 count (in windows of < 100 cells/mm^3^, 100 cells/mm^3^ – 199 cells/mm^3^, and ≥ 200 cells/mm^3^), and hospital admission within the same 12-month period. Potential cases and controls were identified from a prior retrospective cohort study^8^ and by screening all requests for *P. jirovecii* laboratory (microscopy or polymerase chain reaction [PCR]) testing, on any respiratory sample, submitted to the National Health Laboratory Service (NHLS) from Cape Town Metro Hospitals from June 2015 to October 2020. These hospitals include tertiary- (Groote Schuur Hospital), regional- (New Somerset Hospital) and district- (Mitchells Plain Hospital, Heideveld Emergency Centre, Victoria Hospital Wynberg) level care. Demographic and clinical data for included records (including laboratory testing data, HIV and antiretroviral therapy [ART] history, co-morbidities and index hospitalisation treatment and outcome) were collected on a password-protected REDCap electronic data tool^9^ hosted at the University of Cape Town, with access restricted to study authors only.

**TABLE 1:** Definitions for pneumocystis pneumonia and non-pneumocystis pneumonia respiratory disease.

Diagnostic category	Definition
Definite PCP (PCP case)	Microscopy-detected *Pneumocystis jirovecii* in any respiratory sample from an HIV-positive adult presenting with any respiratory symptoms, OR PCR-detected *P. jirovecii* in any respiratory sample from an HIV-positive adult and meeting criteria for probable PCP.
Probable PCP^12,13^	In adults without microscopy or PCR-detected *P. jirovecii*: Clinical syndrome of (1) exertional dyspnoea or non-productive cough, (2) onset within the last 3 months, and (3) tachypnoea, PLUS evidence of diffuse bilateral infiltrates on CXR, OR Decision by treating clinicians to initiate empiric treatment for PCP.
Non-PCP respiratory disease (non-PCP control)	In adults presenting with any respiratory symptom (including cough or dyspnoea with/without chest pain) with negative laboratory tests for *P. jirovecii* and not meeting criteria for probable PCP, with: Alternative aetiology found (laboratory-confirmed), AND/OR PCP-specific treatment not received.

Note: Please see the full reference list of Wills NK, Tavares J, Said-Hartley Q, Wasserman S. Radiological predictors of PCP in HIV-positive adults in South Africa: A matched case-control study (RadPredict). S Afr J HIV Med. 2024;25(1), a1636. https://doi.org/10.4102/sajhivmed.v25i1.1636.

CXR, chest X-ray; PCP, pneumocystis pneumonia; PCR, polymerase chain reaction.

### Definitions

Hypoxia was defined as (1) pulse oximetry (SpO_2_) < 90% on room air, and (2) arterial partial pressure of oxygen (PaO_2_) of < 7.8 kPa on room air, or (3) ratio of arterial partial pressure of oxygen to fraction of inspired oxygen (PaO_2_: FiO_2_, PF ratio) ≤ 300 mmHg^10,11^ on admission. Severe PCP was defined as (1) severe admission hypoxia (PF ratio < 100), (2) patients referred or admitted to ICU, and (3) in-hospital death. PF ratio was imputed for patients with only SpO_2_ (i.e., without PaO_2_) data (available at: https://opencriticalcare.org/imputed-pao2-calculator/).

HIV-positive status required a documented positive HIV enzyme linked immunosorbent assay (ELISA) or any detectable viral load result prior to admission. Patients were recorded to have a smoking history if currently smoking, a five pack-year history or more, or abstained for less than 2 years. Chronic lung disease was defined as post-pulmonary tuberculosis or other reported structural lung disease, including history of smoking or polysubstance induced chronic obstructive pulmonary disease (COPD).

Additional respiratory or systemic pathologies detected on investigation and treated during hospital admission were based on clinician assessment. These included confirmed diagnoses (laboratory-isolated pathogen with pathogen-specific treatment administered, histology-confirmed malignancy or radiologically confirmed pulmonary embolus or pneumothorax in symptomatic patient) or empiric diagnoses (clinical diagnosis assigned and treatment administered by managing clinicians in absence of laboratory or radiological confirmation).

### Radiology review

Admission CXRs were retrieved electronically and reviewed by a specialist radiologist blinded to all clinical and laboratory data. Chest X-rays were systematically analysed using a standardised assessment tool (Online Appendix 1) adapted from the Chest Radiograph Reading and Recording system,^14^ incorporating features identified in the literature to have discriminatory value in distinguishing PCP from non-PCP respiratory disease,^15,16,17,18,19,20,21,22^ with use of descriptive terminology^23^ to enable future interpretation by non-specialist readers, broadening study generalisability.

### Data analysis

Demographic, admission clinical and radiological characteristics in cases and controls were compared through generating proportions as well as crude and adjusted odds ratios (aOR) using logistic regression for categorical variables and using the Chi-squared test for significance testing. Medians and median differences were generated for continuous variables, with use of the Wilcoxon rank-sum for significance testing.

An exploratory radiographic severity score was developed based on evidence from studies using similar scores and correlating pattern of parenchymal abnormality and extent of disease on CXR with PCP prognosis.^24,25,26,27^ Points were allocated as follows:

- for parenchymal pattern:
  ■ no parenchymal abnormalities (1 point)
  ■ reticular or reticulonodular changes (2 points)
  ■ ground-glass opacification or consolidation (3 points)
- extent of disease: 1 point per zone of involvement with any parenchymal changes
- diffuse involvement: 1 point if diffuse descriptor used for any reticular, reticulonodular, ground-glass or consolidation pattern.

For the development of the PCP diagnosis prediction models, based on 52 PCP events, we selected five *a priori* candidate variables^28^: hypoxia (SpO_2_ < 90% in room air, PaO_2_ < 7.8 kPa, or PF ratio ≤ 300 mmHg) or elevated respiratory rate (≥ 30 breaths per minute [bpm]), ground-glass opacification (diffuse or patchy), consolidation, reticular or reticulonodular changes and/or pleural effusion on CXR.^4,5,6,7,21^

For the severe PCP model, we explored parenchymal changes (reticular or reticulonodular changes, ground-glass opacification and consolidation), hypoxia or elevated respiratory rate, and either radiographic severity score or total zones of involvement as candidate variables. We explored a second prognostic model with selection of ground-glass changes, consolidation, respiratory co-diagnosis, and total zones of involvement as candidate variables.

Log transformation of continuous variables with testing of restricted cubic splines to improve model fit, where linear relationship with severe PCP was not displayed, was employed. A backward stepwise approach using the Akaike information criterion (AIC) as the stopping rule was used to select the most predictive variables for the binary logistic regression model.^29^ Model validation was performed with the Houwelingen-Le Cessie heuristic shrinkage estimate and partial residual plots were visually assessed as well as conducting VIF (Variance Inflation Factor) assessment for collinearity, with further internal validation using 200 bootstrap re-samples. Discriminatory performance of the models was assessed using the area under the receiver operating area under the curve (AUC) or equivalent c (concordance) index. Analyses were performed in RStudio (version 4.3.1).

### Ethical considerations

This study was approved by the University of Cape Town Human Research Ethics Committee (reference no. 522/2019; parent study reference no. 548/2015) which waived the requirement for informed consent. Hospital approval was received from facilities and the National Health Research Database (NHRD). Data retrieval from the NHLS was approved via the Academic Affairs and Research Management System (AARMS). The study was conducted in accordance with the Helsinki Declaration as revised in 2013.

## Results

**FIGURE 1:**
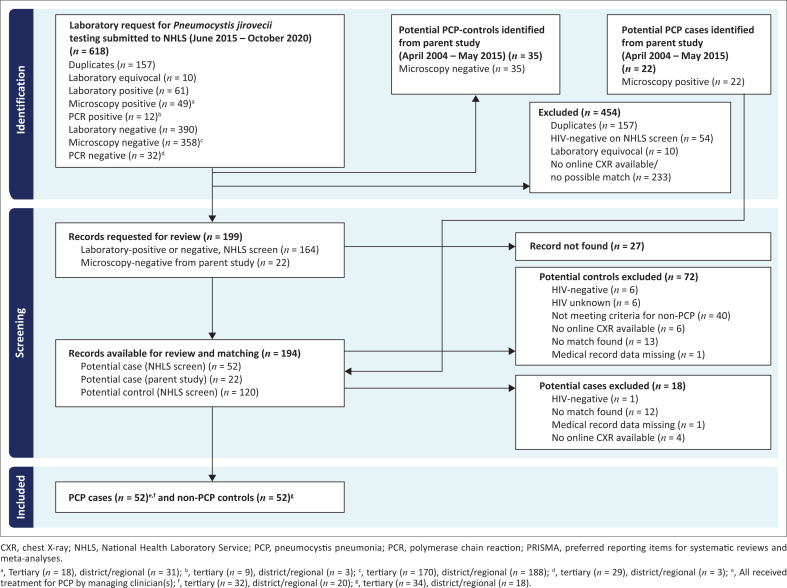
PRISMA diagram: Flow of records from National Health Laboratory Service and parent study screen through to final pneumocystis pneumonia cases and non-pneumocystis pneumonia control inclusion.

Fifty-two cases with definite PCP and 52 controls with non-PCP respiratory disease were included (Figure 1).

A higher proportion of cases compared to controls were newly diagnosed with HIV on admission (38% versus 21%). Cotrimoxazole prophylaxis exposure was low across both groups. Chronic lung disease and previous pulmonary tuberculosis were more frequently reported in controls. Cases had more respiratory distress on admission compared with controls, with higher median respiratory rate (median 34 versus 28 bpm) and more frequent hypoxia (44% versus 28%). Intensive care unit referral or admission, as well as in-hospital mortality, were similar between the two groups (Table 2). Confirmed and empiric respiratory co-diagnoses are outlined in Online Appendix 1, Figure 1-A1. Within cases, frequent co-diagnoses included bacterial pneumonia (46%), viral pneumonia (15%) and pulmonary or disseminated tuberculosis (10%). Within controls, primary diagnoses included bacterial pneumonia (67%), pulmonary or disseminated pulmonary tuberculosis (46%), acute exacerbation of COPD (12%), pulmonary oedema (10%) and viral pneumonia (4%).

**TABLE 2:** Comparison of demographic, admission and outcome characteristics of HIV-positive adults with pneumocystis pneumonia (cases) versus non-pneumocystis pneumonia respiratory disease (controls).

Variable	Category	PCP (*n* = 52)					Non-PCP (*n* = 52)					*P*
		*n*	%	Median	Range	IQR	*n*	%	Median	Range	IQR	
**Demographics and HIV history**												
Female gender	-	36	69	-	-	-	31	60	-		-	0.307
Age (median years)	-	-	-	34.5	-	29–42	-	-	38	-	31–42	0.342
Newly diagnosed with HIV on admission	-	20	38.4^a^	-	-	-	11	21.2	-	-	-	0.048
Cotrimoxazole prophylaxis use on admission	Yes	4	7.7	-	-	-	5	9.6	-	-	-	0.782
	No	36	69.2	-	-	-	37	71.2	-	-	-	0.782
	Not reported	12	23.1	-	-	-	10	19.2	-	-	-	0.667
ART history	ART naïve	27	52.9^a^	-	-	-	21	41.1^a^	-	-	-	
	Currently on ART	10	19.6^a^	-	-	-	8	15.7^a^	-	-	-	0.911
	Interrupted ART	14	27.5^a^	-	-	-	22	43.1^a^	-	-	-	0.110
	VL undetectable	1	1.9	-	-	-	3	5.8	-	-	-	0.331
	Median VL (copies/ mm^3^)	-	-	125 672	-	18 872–341 875^b^	-	-	53 335	-	8782–206 702^b^	0.395
Median CD4 count (cells/mm^3^)	-	-	-	21	2–405	10–47	-	-	40	4–461	20–79	0.011
Other co-morbidities	Chronic lung disease	6	11.5	-	-	-	20	38.5	-	-	-	0.003
	Previous pulmonary tuberculosis	17	34^c^	-	-	-	26	51^a^	-	-	-	0.086
**Admission details**												
Level of care	District/regional	18	34.6	-	-	-	18	34.6	-	-	-	-
	Tertiary	34	65.4	-	-	-	34	65.4	-	-	-	1.000
Admission respiratory rate (median bpm)	-	-	-	34	-	28–38	-	-	28	-	22–32	0.003
Hypoxia on admission^d^	-	44	88^c^	-	-	-	28	53.8	-	-	-	0.000
Admission PF ratio (median mmHg)^e^	-	-	-	218.5	-	164–269	-	-	296	-	199.5–346.5	0.007
Haemoglobin (median g/dL)	-	-	-	11.0	-	9.7–12.4	-	-	10.4	-	8.7–11.7	0.080
White cell count (median × 10^9^ cells/L)	-	-	-	8.4	-	5.7–12.5	-	-	7.3	-	4.9–13.6	0.333
**Outcomes**												
ICU referral	-	18	34.6	-	-	-	21	40.4	-	-	-	0.544
ICU admission	-	16	30.8	-	-	-	20	38.5	-	-	-	0.410
Mechanically ventilated	-	16	30.8	-	-	-	20	38.5	-	-	-	0.410
Inotrope support	-	6	11.5	-	-	-	13	25	-	-	-	0.082
In-hospital death	-	19	36.5	-	-	-	16	30.8	-	-	-	0.534
ICU death	-	7	43.8^f^	-	-	-	12	60^f^	-	-	-	0.433

Note: Denominator: *n* = 52 unless specified.

ART, antiretroviral therapy; bpm, breaths per minute; ICU, intensive care unit; IQR, interquartile range; PaO_2_, partial pressure of oxygen in arterial blood; PCP, pneumocystis pneumonia; PF, PaO_2_:FiO_2_ ratio (partial pressure of oxygen in arterial blood: inspired oxygen concentration ratio); RA, room air; SpO_2_, pulse oximetry saturation; VL, viral load.

a , denominator = 51;

b , denominator = 14 (adults with available and detectable recent VL);

c , denominator = 50;

d , SpO_2_ < 90% (RA) or PaO2 < 7.8 kPa (RA) or PF ratio ≤ 300 mmHg;

e , imputed PF ratio: to allow standardised assessment of PF ratio trend, an imputed PF ratio was calculated for patients with only SpO_2_ available on oxygen or room air (*n* = 39) (available at: https://opencriticalcare.org/imputed-pao2-calculator/);

f , denominator = adults admitted to ICU.

### Chest X-ray features associated with pneumocystis pneumonia

Admission CXR quality was assessed as optimal for 69% of radiographs; suboptimal CXR quality was mainly due to poor lung expansion or inadequate inspiration (20%) and/or poor patient positioning (14%) (Online Appendix 1, Table 1-A1).

One case and one control patient had a normal CXR. Three control patients (5.8%), and no cases, had a pneumothorax on admission CXR (five patients overall, three of whom had PCP, subsequently developed pneumothorax as related to mechanical ventilation or a procedure). Cystic lesions were only seen in two control patients. Parenchymal calcification was seen in 7 (13.5%) control patients and in none of the PCP cases (Online Appendix 1, Table 2-A1).

**FIGURE 2:**
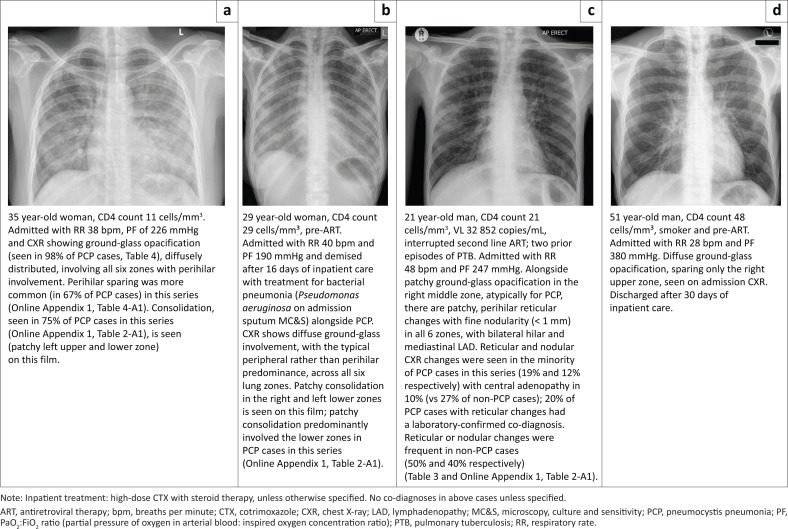
(a–d) Selected chest X-rays from HIV-positive adults with pneumocystis pneumonia.

**FIGURE 3:**
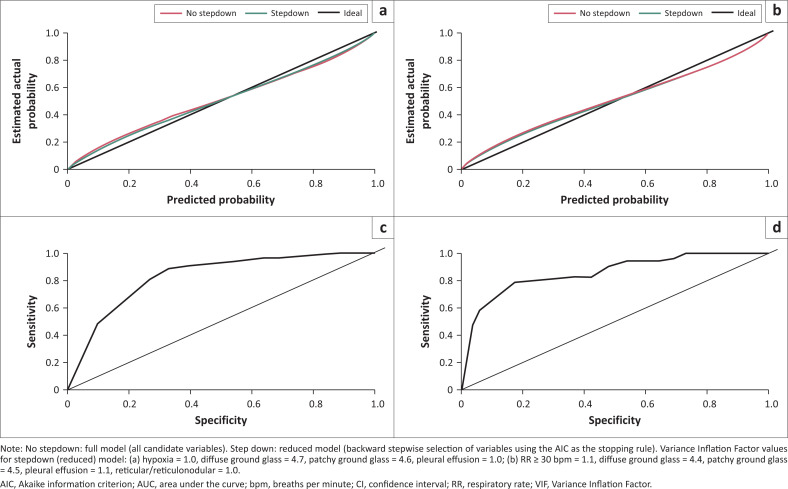
Calibration curve of the multivariable logistic regression hypoxia model (a) and respiratory rate model (b), comparing actual probability to model-predicted probability (for the full [no stepdown] and reduced [stepdown] models), for the diagnosis of pneumocystis pneumonia in HIV-positive adults, with receiver operating characteristic and area under the curve for the (c) hypoxia (AUC: 0.828, 95% CI: 0.750–0.906) and (d) respiratory rate models (AUC: 0.857, 95% CI: 0.786–0.928).

Diffuse ground-glass opacification was associated with significantly increased odds of PCP on adjusted analysis (aOR 6.2, 95% confidence interval [CI]: 1.6–28.9, *P* = 0.01, Table 3). Consolidation was frequently seen on CXR in both cases and controls, but patchy compared to diffuse consolidation was associated with increased odds of PCP (aOR 5.8, 95% CI 1.1–45.7, *P* = 0.05; Online Appendix 1, Table 3-A1 and Online Appendix Figure 2-A1). In contrast, pleural effusion was associated with decreased odds of PCP (aOR 0.1, 95% CI 0.0–0.4, *P* = 0.01), as were reticular or reticulonodular abnormalities, cavitation and central lymphadenopathy, although these were not statistically significant (Table 3). There was no difference in the median radiographic severity score in cases (median: 10, range: 3–12) compared to controls (median: 10, range: 1–12), with no relationship on adjusted analysis (OR: 1.3 for one-unit score increase, 95% CI: 0.9–2.2, *P* = 0.2). Selected examples of typical CXRs with notable features from cases with PCP in this series are shown in Figure 2 and Online Appendix 1, Figure 3-A1.

**TABLE 3:** Chest X-ray features in cases compared controls.

CXR feature	PCP (*n* = 52)		Non-PCP (*n* = 52)		Crude		Adjusted		*P*
	*n*	%	*n*	%	OR	95% CI	OR†	95% CI	
**Parenchymal change**									
Reticular ± nodular	10	19.2	26	50	0.2	0.1–0.6	0.4	0.1–1.4	0.118
Reticulonodular	6	11.5	21	40.4	0.2	0.1–0.5	0.2	0.0–1.1	0.086
Diffuse ground-glass opacities	44	86.3	22	52.4	5.7	2.2–16.5	6.2	1.6–28.9	0.011
Patchy ground-glass opacities	7	13.7	20	47.6	0.2	0.1–0.5	0.2	0.0–0.6	0.011
Consolidation	39	75	32	61.5	1.9	0.8–4.4	1.9	0.4–8.0	0.396
Cavitation	3	5.8	9	17.3	0.3	0.1–1.1	1.4	0.1–20.6	0.822
**Other**									
Pleural effusion	2	3.8	14	26.9	0.1	0.0–0.4	0.1	0.0–0.4	0.011
Central lymphadenopathy	5	9.6	14	26.9	0.3	0.1–0.8	0.4	0.1–1.7	0.206

CI, confidence interval; CXR, chest X-ray; OR, odds ratio; PCP, pneumocystis pneumonia; PF, PaO_2_:FiO_2_ ratio (partial pressure of oxygen in arterial blood: inspired oxygen concentration ratio).

† , Adjusted for chronic lung disease and PF ratio.

### Clinical prediction model for pneumocystis pneumonia diagnosis

Variables selected *a priori* for inclusion in the diagnostic model were hypoxia (SpO_2_ < 90% in room air, PaO_2_ < 7.8 kPa, or PF ratio ≤ 300 mmHg) or elevated respiratory rate (≥ 30 bpm ground-glass opacification, diffuse or patchy), consolidation, reticular or reticulonodular changes and/or pleural effusion on CXR.^4,5,6,7,21^ After backward stepwise selection using the AIC as the stopping rule, the following variables were included in the reduced binary regression models: (1) for the hypoxia model: hypoxia, diffuse or patchy ground-glass opacification, and pleural effusion, and (2) for the respiratory rate model: respiratory rate ≥ 30 bpm, diffuse or patchy ground-glass opacification, pleural effusion and reticular or reticulonodular changes (Table 4). Both models showed good calibration – the Houwelingen-Le Cessie heuristic shrinkage estimate for the hypoxia model was 0.14, and for the respiratory rate model, 0.13, suggesting only a marginal (14% and 13% respectively) decrease in performance can be expected with model testing on new data). Partial residual plots showed no collinearity with VIF close to 1 (indicating marginal inter-variable correlation, and therefore predictor regression coefficients are likely reliable and not inflated due to interactions with other predictors) (Figure 3a and 3b). Regression coefficients for the full (all candidates) and reduced models (selected candidates) for the hypoxia and respiratory rate model are shown in Table 4.

**TABLE 4:** Multivariable model of predictors of pneumocystis pneumonia – Hypoxia and respiratory rate model.

Variable	Full model			Reduced model				*P*
	Regression coefficient	SE	*P*	Regression coefficient	SE	OR	95% CI	
**Hypoxia model**								
Hypoxia†	0.8	0.5	0.1	1.0	0.5	2.8	1.1–7.5	0.036
Diffuse ground-glass opacification	2.9	1.1	0.01	3.0	1.1	20.6	2.3–183.8	0.006
Patchy ground-glass opacification	1.2	1.2	0.3	1.2	1.2	3.3	0.3–32.9	0.326
Pleural effusion	−2.6	0.9	0.003	−2.6	0.9	0.1	0.0–0.4	0.002
Reticular ± nodular changes‡	−1.0	0.5	0.1	-	-	-	-	-
Consolidation‡	−0.1	0.6	0.9	-	-	-	-	-
**Respiratory rate model**								
Respiratory rate ≥ 30 bpm	1.3	0.5	0.02	1.3	0.5	3.5	1.3–9.8	0.017
Diffuse ground-glass opacification	2.6	1.1	0.02	2.6	1.1	13.6	1.5–123.5	0.020
Patchy ground-glass opacification	0.8	1.2	0.5	0.8	1.2	2.1	0.2–2.1	0.521
Pleural effusion	−2.7	0.9	0.002	−2.7	0.9	0.1	0.0–0.4	0.002
Reticular ± nodular changes	−1.1	0.6	0.04	−1.1	0.5	0.3	0.1–0.9	0.040
Consolidation‡	−0.03	0.6	1.0	-	-	-	-	-

Note: C index hypoxia model: full model = 0.843, reduced model = 0.828. C index respiratory rate model: full model = 0.859, reduced model = 0.857.

bpm, breaths per minute; OR, odds ratio; PaO_2_, partial pressure of oxygen in arterial blood; PCP, pneumocystis pneumonia; PF, PaO_2_:FiO_2_ ratio (partial pressure of oxygen in arterial blood: inspired oxygen concentration ratio); RA, room air; RR, respiratory rate; SpO_2_, pulse oximetry saturation; SE, standard error.

† , SpO_2_ < 90% (RA) or PaO_2_ < 7.8 kPa (RA) or PF ratio ≤ 300 mmHg;

‡ , dropped from model on backwards stepwise regression using the Akaike information criterion (AIC) as a stopping rule.

The AUC of the receiver operating characteristic (ROC) was 0.828 (95% CI: 0.750–0.906) (Figure 3c) for the hypoxia model and 0.857 (95% CI: 0.786 – 0.928) (Figure 3d) for the respiratory model (using the DeLong method on bootstrap validation samples). Additional probability and validation plots are included in Online Appendix 1, Figure 4-A1 and Online Appendix, Figure 5-A1. A proposed algorithm for the diagnosis of PCP using admission clinical parameters and CXR features has been outlined in Online Appendix 1, Figure 6-A1.

### Chest X-ray changes associated with severe pneumocystis pneumonia

Twenty-nine cases met criteria for severe PCP disease (PF ratio < 100 mmHg [*n* = 3], ICU referral or admission [*n* = 18] or in-hospital death [*n* = 19]). Median PF ratio was lower among patients with severe versus non-severe PCP (189 mmHg [IQR: 129–192] versus 247 mmHg [IQR: 208–270]).

Diffuse, compared to patchy, ground-glass opacification was associated with increased odds of severe PCP (aOR: 4.5, 95% CI: 1.6–14.4, *P* = 0.008) as was upper zone involvement (aOR: 5.9, 95% CI: 2.0–20.7, *P* = 0.003) (Online Appendix 1, Table 4-A1 and Online Appendix 1, Figure 7-A1). The odds of severe PCP increased with increasing ground-glass zone involvement (aOR: 2.1 for each one-unit increase in involved zone; 95% CI: 1.4–3.2, *P* = 0.0004). Consolidation was strongly associated with severe PCP (aOR: 3.3, 95% CI: 1.2–11.0, *P* = 0.03), with higher odds for severe disease with patchy rather than diffuse involvement (aOR: 5.5, 95% CI: 1.2–39.3, *P* = 0.04) and perihilar sparing (aOR: 3.9, 95% CI: 1.2–13.5, *P* = 0.04). Increasing zones of involvement with consolidation did not correlate with severe PCP. Increasing radiographic score was not predictive of severe PCP (aOR: 1.08, 95% CI: 0.8 – 1.4, *P* =0.6 for each point increase).

### Clinical prediction model for severe pneumocystis pneumonia

None of the selected candidate variables for the first model (reticular/reticulonodular changes, ground-glass opacification, consolidation, with either elevated respiratory rate or hypoxia, and either radiographic severity score or total zones of involvement) or second model (with candidate variables for the first model, but omitting reticular or reticulonodular and including respiratory co-diagnosis) met the model inclusion threshold using the AIC stopping rule.

## Discussion

In this matched case-control study involving 104 HIV-positive adults in South Africa, diffuse ground-glass changes, predominantly with perihilar sparing, were significantly associated with HIV-associated PCP. Pleural effusion had a strong negative correlation with PCP, and alongside reticulonodular changes, was seen with higher frequency in non-PCP disease. Two regression models, incorporating either hypoxia or elevated respiratory rate, with diffuse ground-glass changes, absence of pleural effusion or absence of reticular/reticulonodular changes, performed well in discriminating PCP from non-PCP respiratory disease in this population.

The hazy shadowing of ground-glass changes seen in PCP are a reflection of the exuberant host inflammatory response that is triggered by *P. jirovecii* attachment to alveolar pneumocytes and extracellular matrix proteins,^30^ with resultant interstitial thickening, partial alveolar exudative filling, air displacement and/or alveolar collapse.^23^ The neutrophil- and CD8^+^-driven immune reaction that incites lung injury and contributes to respiratory failure in PCP^31^ occurs paradoxically in patients with advanced immunodeficiencies. In this study, a significant proportion of adults with PCP were profoundly immunosuppressed (77% of adults had CD4 count < 50 cells/mm^3^); concordantly, hypoxia was seen in 88% of PCP cases.

Reticular changes, inferring net-like interlobular septal thickening that may coalesce into nodules but characteristically spare the airspace,^23^ as well as pleural disease, were not associated with PCP in our study as in others^5,32,33,34^ and strongly suggest an alternative non-PCP diagnosis. This is consistent with pathology induced by tropism of *P. jirovecii* for alveolar epithelium (airspace opacification). No patients with PCP had pneumothorax or cystic changes on admission CXR in this study. Previous commentaries, largely skewed by retrospective reviews of all cases of HIV-associated pneumothorax^35,36^ and historical reports linking pneumothorax to pentamidine prophylaxis failure with progressive upper zone fibrocystic disease,^37^ may have over-represented true and contemporary rates of pneumothorax amongst adults with PCP. Three patients in our PCP cohort developed ventilation-associated pneumothorax, a complication attributed to the pathological reduction in alveolar surfactant seen as a consequence of the *P. jirovecii* immune response, that reduces lung compliance and increases risk of alveolar rupture.^38^ Central lymphadenopathy correlated more strongly with non-PCP respiratory disease in our study, and is a well-described radiographic feature in many alternative HIV-associated pathologies including tuberculosis, fungal infections and lymphoma.^39,40^

Severe PCP was seen in more than half (56%) of PCP cases. Poor outcomes in adults with PCP are thought to be, in part, a consequence of dysregulated host immune response, in keeping with the mortality benefit demonstrated with addition of corticosteroids in treating severe PCP,^41^ and the correlation between poor outcomes and more extensive CXR involvement,^24,25,27^ higher serological indices of inflammation and hypoxia.^8,25^ In keeping with these observations, diffuse ground-glass opacification was associated with severe PCP in our study. There was a shift to increasing zone (particularly with ground-glass opacities) and diffuse CXR involvement in severe disease. Consolidation was a frequent feature in both PCP cases (75%) and in non-PCP controls (62%). In keeping with prior studies showing radiological progression from interstitial to alveolar infiltrates correlating with worsening clinical PCP severity,^24,25^ consolidation had predictive value for severe PCP disease in our study.

Prior studies have examined a combination of clinical and radiological variables for PCP prediction^5,6,7^ but have had limited power and/or discriminatory value and therefore not easily translated into tools that can enhance clinical decision-making at the bedside. A clinical prediction rule developed by Maartens et al.,^4^ based on 29 microscopy-confirmed PCP cases with imputation to a total of 56 events, incorporated CXR changes (possible or likely PCP), haemoglobin ≥ 9 g/dL and either elevated respiratory rate or low pulse oximetry saturation for predicting PCP; both models performed well with receiver operative characteristic AUCs of at least 0.8. In our study, we interrogated specific and descriptive CXR features that offer discriminatory value for predicting PCP versus other common HIV-associated respiratory diseases, to develop prediction models. Using either presence of hypoxia or elevated respiratory rate, together with selected CXR features, these models had robust internal validation and good discriminatory performance (both with AUC greater than 0.82). In contrast to the Maartens study, we did not find a correlation between haemoglobin and PCP diagnosis. In our study, greater representation of critically ill adults across both comparator groups, with multiple overlapping co-diagnoses and comparatively lower median CD4 count in both PCP cases and controls compared to the Maartens cohort, may possibly explain the absence of this correlation.

Despite our use of a broad definition for PCP severity and high representation of severe cases in our cohort, none of the selected candidate variables for severe PCP was sufficiently predictive and we were not able to generate a prognostic model for PCP. Our cross-sectional analysis of admission CXRs did not capture CXR evolution over time and after exposure to PCP-directed therapy; there is some evidence linking CXR progression over time, rather than baseline features, to the need for incremental mechanical ventilation support and poor outcome in ICU settings.^42^ A study from China, enrolling 1001 adults with HIV-associated PCP and a 17% in-hospital mortality rate, found a six-variable predictive model incorporating elevated lactate dehydrogenase (LDH), hypoxia, ICU admission, anaemia, low CD4 count and development of post-admission pneumothorax to offer good discriminatory value for predicting in-hospital death (AUC 0.9).^43^ Other studies have found increasing age,^44^ hypoxia,^8,24,44,45^ LDH,^8,46^ concomitant comorbidity or coinfection,^8,44,47,48^ high, Sequential Organ Failure Assessment (SOFA), or Acute Physiology and Chronic Health Evaluation (APACHE) scores,^48,49^ acidosis^42^ and incremental ventilatory support requirement^42,48^ to be associated with poor outcome. This wide between-study variability in markers of severe PCP highlight the challenges with developing generalisable and reproducible prognostic rules for PCP, and may indicate differing clinical phenotypes and gaps in our current understanding of the pathophysiology of severe disease and therefore its clinical or radiological correlates.

Our study has some limitations. By virtue of its retrospective design, our findings are limited by the reliance on laboratory specimen submission, accuracy of medical records and quality of radiographs. Selection bias may have been introduced since patients with suggestive clinical or radiological features of PCP may have received empiric treatment without pursuing laboratory confirmation, with possibly higher representation of those with less typical presentations. Furthermore, critically ill (and non-ventilated) adults, unable to produce sputum or undergo invasive respiratory sampling, who may represent a distinct clinical and radiological phenotype, would not be represented in this study. Whilst adults with negative *P. jirovecii* laboratory testing, but meeting the criteria for probable PCP, were excluded to strengthen the confidence in the definite PCP versus non-PCP comparative analysis, inclusion of this subgroup may have added power to a predictive model for any (definite or probable) PCP or severe PCP. Our predictive model performed well on internal validation but requires evaluation in a separate cohort to confirm external validity. Although clinician-assigned co-diagnoses were captured and adjusted for in the severe PCP analysis (a recent study showed increased mortality with pulmonary tuberculosis coinfection),^8^ this could not be done in the PCP diagnosis models as the control group required an alternate primary, non-PCP, diagnosis.

## Conculsion

Our study identified CXR changes that correlate with laboratory-confirmed PCP and that can be utilised together with objective, easily obtainable clinical information for accurate and prompt PCP recognition. These findings may be used to train evolving artificial intelligence (AI)-assisted CXR reading software,^50,51^ offering potential value in settings where access to specialist radiologist services are limited. Other non-sputum-based diagnostics, such as serum (1–3)-β-D-Glucan which has good sensitivity for PCP diagnosis,^52^ may further enhance performance of diagnostic algorithms incorporating clinical and radiological predictors, and should be explored in high-burden settings where access to sputum-based diagnostics for PCP is limited.
